# Outcomes among HIV-1 Infected Individuals First Starting Antiretroviral Therapy with Concurrent Active TB or Other AIDS-Defining Disease

**DOI:** 10.1371/journal.pone.0083643

**Published:** 2013-12-31

**Authors:** André R. S. Périssé, Laura Smeaton, Yun Chen, Alberto La Rosa, Ann Walawander, Apsara Nair, Beatriz Grinsztejn, Breno Santos, Cecilia Kanyama, James Hakim, Mulinda Nyirenda, Nagalingeswaran Kumarasamy, Umesh G. Lalloo, Timothy Flanigan, Thomas B. Campbell, Michael D. Hughes, on behalf of the P E A R L S study team of the ACTG

**Affiliations:** 1 Departamento de Ciências Biológicas, Escola Nacional de Saúde Pública Sergio Arouca, Fundação Oswaldo Cruz, Rio de Janeiro, Brazil; 2 Center for Biostatistics in AIDS Research, Harvard School of Public Health, Boston, Massachusetts, United States of America; 3 Asociacion Civil Impacta Salud y Educacion - Barranco, Lima, Peru; 4 Frontier Science and Technology Research Foundation, Amherst, New York, United States of America; 5 Evandro Chagas Clinical Research Institute, Fiocruz, Rio de Janeiro, Brazil; 6 Hospital Nossa Senhora da Conceição, Porto Alegre, Brazil; 7 Kamuzu Central Hospital, Lilongwe, Malawi; 8 University of Zimbabwe College of Health Sciences, Harare, Zimbabwe; 9 Mulinda Nyirenda, College of Medicine, University of Malawi, Blantyre, Malawi; 10 YRG Centre for AIDS Research and Education, Chennai, India; 11 Nelson R. Mandela School of Medicine, Durban, South Africa; 12 Brown Medical School, Providence, Rhode Island, United States of America; 13 Division of Infectious Diseases, Department of Medicine, University of Colorado School of Medicine, Aurora, Colorado, United States of America; 14 Department of Biostatistics, Harvard School of Public Health, Boston, Massachusetts, United States of America; Johns Hopkins University School of Medicine, United States of America

## Abstract

**Background:**

Tuberculosis (TB) is common among HIV-infected individuals in many resource-limited countries and has been associated with poor survival. We evaluated morbidity and mortality among individuals first starting antiretroviral therapy (ART) with concurrent active TB or other AIDS-defining disease using data from the “Prospective Evaluation of Antiretrovirals in Resource-Limited Settings” (PEARLS) study.

**Methods:**

Participants were categorized retrospectively into three groups according to presence of active confirmed or presumptive disease at ART initiation: those with pulmonary and/or extrapulmonary TB (“TB” group), those with other non-TB AIDS-defining disease (“other disease”), or those without concurrent TB or other AIDS-defining disease (“no disease”). Primary outcome was time to the first of virologic failure, HIV disease progression or death. Since the groups differed in characteristics, proportional hazard models were used to compare the hazard of the primary outcome among study groups, adjusting for age, sex, country, screening CD4 count, baseline viral load and ART regimen.

**Results:**

31 of 102 participants (30%) in the “TB” group, 11 of 56 (20%) in the “other disease” group, and 287 of 1413 (20%) in the “no disease” group experienced a primary outcome event (p = 0.042). This difference reflected higher mortality in the TB group: 15 (15%), 0 (0%) and 41 (3%) participants died, respectively (p<0.001). The adjusted hazard ratio comparing the “TB” and “no disease” groups was 1.39 (95% confidence interval: 0.93–2.10; p = 0.11) for the primary outcome and 3.41 (1.72–6.75; p<0.001) for death.

**Conclusions:**

Active TB at ART initiation was associated with increased risk of mortality in HIV-1 infected patients.

## Introduction

The benefits of highly active antiretroviral therapy (ART) are unequivocal and their widespread use in developed countries has reduced HIV-associated morbidity and mortality [1]–[3]. Approximately 30–60% of all newly diagnosed HIV-infected persons present to clinics with either an AIDS-defining disease or a CD4+ cell count lower than 350 cells/mm^3^ [4], [5]. Despite effective viral suppression and reduced HIV-associated morbidity and mortality among these persons, there may be less than ideal immune reconstitution of CD4 cells, higher incidence of immune reconstitution inflammatory syndrome (IRIS), and interactions between antiretroviral therapy (ART) regimens and drugs used to treat opportunistic infections (OI) [6]–[8]. Mortality rates subsequent to an episode of AIDS-defining disease may depend on the specific disease [9].

Tuberculosis (TB) is the most common AIDS-defining disease in several resource-limited countries (RLC) [10], [11]. A recent meta-analysis of cohort studies has shown that TB was associated with poor survival among HIV-infected individuals not receiving ART but not among those receiving ART [11]. However, response to ART may be related to timing of ART initiation during TB treatment [12]–[14] and CD4+ cell count at treatment initiation [15]. The World Health Organization (WHO) recommends start of ART as soon as possible in all HIV/TB co-infected patients with active TB and within the first eight weeks of starting TB treatment [16]. The Centers for Disease Control and Prevention (CDC) recommendation varies from 2 to 4 weeks depending on CD4 count and clinical setting [17]. Current evidence indicates a benefit for starting ART within the first 2 weeks of treatment for *Pneumocystis jirovecii* pneumonia (PCP) and serious bacterial infections [18]. Less information is available for other non-TB AIDS-defining diseases and no formal recommendation is made, except for ART initiation as soon as the clinical condition allows, mainly for diseases in which a specific treatment is not available and ART is the current recommended treatment [16], [18], [19].

The aim of this report is to investigate the impact of ART on morbidity (time to AIDS-defining diseases and virologic failure) and mortality (all-cause death) for ART-naïve HIV-1-infected patients presenting with a diagnosis of TB or other AIDS-defining disease at study entry compared to those presenting without any ongoing (active) AIDS-defining disease in the setting of an international multicenter ART trial in RLC [20].

## Materials and Methods

The AIDS Clinical Trials Group (ACTG) Prospective Evaluation of Antiretrovirals in Resource- Limited Settings (PEARLS, also known as ACTG A5175; ClinicalTrials.gov NCT00084136) study was a phase IV, prospective, randomized, open-label evaluation of the efficacy of once-daily protease inhibitor and once-daily non-nucleoside reverse transcriptase inhibitor antiretroviral combinations for the initial treatment of HIV-1-infected individuals from nine countries in Africa, Asia, the Caribbean, and North and South America. The participants were ≥18 years old, had documented HIV-1 infection, had received no more than 7 days of cumulative prior ART (prior use of single-dose nevirapine or zidovudine for any duration to prevent mother-to-child transmission of HIV was allowed), and had a CD4 cell count <300 cells/mm^3^ within 90 days prior to entry into the study. All participants had baseline clinical assessments for co-morbid conditions and those with an AIDS-defining disease were allowed to enroll only if stable and having already completed at least 14 days of treatment for that condition, if applicable. Participants were randomized to one of the following arms: A, lamivudine/zidovudine+efavirenz (3TC/ZDV+EFV); B, didanosine_EC+emtricitabine+atazanavir (DDI-EC+FTC+ATV); C, emtricitabine/tenofovir-DF+efavirenz (FTC/TDF+EFV). Visits were scheduled at screening, entry, and weeks 2, 4, 8, 12, 16, 20, 24, and every 8 weeks thereafter; follow-up continued for the duration of the study irrespective of changes in antiretroviral therapy. Enrollment occurred between May 2005 and July 2007, and participants were followed through to April/May 2010. Further details about this study are reported elsewhere [20].

Diagnosis of TB and AIDS-defining diseases at all study locations used standard ACTG definitions, including categorization as either confirmed or presumptive [20]. A panel of five physician team members, who were blinded to participant identity, clinic site, demographic characteristics and study treatment, then reviewed the supporting clinical and laboratory data for each diagnosis and made a final classification of each diagnosis according to these definitions. For analysis purposes, participants were categorized retrospectively into one of three study groups based on the presence or not of an active confirmed or presumptive disease at the time of ART initiation: (1) those with a concurrent diagnosis of pulmonary and/or extra pulmonary TB – PTB/ETB (the “TB” group), (2) those with any other concurrent AIDS-defining disease (the “other AIDS-defining disease” group), or (3) those without TB or other AIDS-defining disease (the “no disease” group).

The study’s primary outcome (and the outcome of interest in this report), was the time from randomization to the first of any of the following events: virologic failure – defined as plasma HIV-1 RNA ≥1000 copies/mL on two consecutive measurements obtained at the week 16 visit or later; disease progression – defined as a new or recurrent AIDS-defining OI or malignancy occurring at least 12 weeks following randomization and not associated with a diagnosis of IRIS (Immune Reconstitution Inflammatory Syndrome); or death due to any cause. If a participant failed to attend a scheduled follow-up visit, sites were required to assess the reason for missed visit. If death was the reason for a missed visit, sites were required to report the date of death, the suspected cause of death, relationship to study treatment and the sources of death-related information.

Differences in age, sex, country, screening CD4 count, baseline viral load (VL, measured by plasma HIV-1 RNA level) and chance imbalance by randomized ART regimen among the three baseline disease status groups (“TB”, “other AIDS-defining disease” and “no disease”) were evaluated using the chi-square test for categorical variables and analysis of variance for continuous variables. Comparison between groups in time from diagnosis of concurrent disease to randomization used the Wilcoxon Rank-Sum Test. The Kaplan-Meier method estimated, and the log-rank test compared, the cumulative probability of the primary outcome over time among the study groups. Participants lost to follow-up were considered censored at the date of last contact. Cox proportional hazard models compared the relative hazard of the primary outcome, while adjusting for other baseline covariates listed above. To evaluate whether any study group differences varied by assigned ART regimen, a term for the interaction of ART arm and study group was included in the model. Other first order interaction terms were also tested. Similar methods were used to analyze the time to each of the three, separate components of the primary outcome, i.e., time to virologic failure, time to disease progression, and time to death.

On May 6, 2008, the independent Data and Safety Monitoring Board (DSMB) for the study recommended stopping treatment with the once-daily protease inhibitor-based regimen (arm B) due to conclusive evidence of inferiority compared to the control arm (arm A). Study participants, investigators, institutional review boards, and ethics committees were then informed of the DSMB findings and participants still taking ATV+DDI-EC+FTC were switched to alternative antiretroviral regimens and continued follow-up, but assigned study treatment in the other arms continued. In our analysis we used data for the entire follow-up available for each participant. A different approach, censoring the follow-up of participants in arm B in May 2008, gave similar results, and therefore, only results from the former analysis are presented here.

Written informed consent was obtained from all participants, and the human experimentation guidelines of the US Department of Health and Human Services were followed. The study was approved by the Colorado Multiple Institutional Review Board, University of Colorado (USA) and local Ethics Committees at each participating institution in Brazil, Haiti, India, Malawi, Peru, South Africa, Thailand and Zimbabwe.

## Results

Between May 2005 and July 2007, a total of 1,571 HIV-1 positive individuals were enrolled and randomly assigned to one of the three ART arms. One hundred two participants (6.5% of the study population) had concurrent tuberculosis at ART initiation, 83 PTB and 19 ETB. Fifty-six participants (3.6% of the study population) had other AIDS-defining disease (18 mucocutaneous herpes simplex, 10 wasting syndrome, 9 disseminated fungal disease, 5 Kaposi sarcoma, 4 toxoplasmic encephalitis, 3 esophageal candidiasis, 2 cytomegalovirus retinitis, 2 PCP, 1 cryptococcal meningitis, 1 HIV-associated dementia and 1 *Mycobacterium avium* complex). Excluding recurrent and chronic AIDS-defining diseases, the median time from the diagnosis of the concurrent AIDS defining disease to ART initiation was 14 weeks (range, 0–77) for participants in the “TB” group and 8 weeks (range, 1–348) for those in the “other AIDS-defining disease” group (p = 0.002). The remaining 1,413 participants (90% of the study population) did not have a concurrent diagnosis and therefore made up the “no disease” group. The median time of follow-up from randomization to a primary outcome or censoring was 178 weeks (range, 3–241) in the “TB group”, 168 weeks (range, 0–256) in the “other AIDS-defining disease” group, and 176 weeks (range, 0–261) in the “no disease” group. Only 63 of the 1,571 participants (4%) received ART before entering the study. Of those, 57 were in the “no disease” group and only 3 in each of the “TB” and “other AIDS-defining disease” groups.

Except for age and randomized treatment, the selected baseline characteristics of the participants differed significantly among disease groups (Table 1). Participants in the “other AIDS-defining disease” group were more likely to be male (79%) than those in the “TB” group (58%) and “no disease” group (52%). Diseases were distributed unequally among countries: approximately 80% of all TB cases were reported from participants from either India (56%) or South Africa (27%), while approximately 60% of participants with other AIDS-defining diseases were from Peru (27%) and the United States (34%). Seventy-eight percent of participants in the “TB” group and 71% in the “other AIDS-defining disease” group had CD4 count below 200 cells/mm^3^, compared with 59% in the “no disease” group. In fact, 32% of participants in the “other AIDS-defining disease” group had CD4 count below 50 cells/mm^3^ compared with 12% in both the “TB” and “no disease” groups. Seventy-five percent of participants in the “TB” group had HIV-1 RNA level greater than or equal to 100,000 copies/mL compared with 55% in the “other AIDS-defining disease” group and 50% in the “no disease” group.

**Table 1:** Selected baseline characteristics by study groups.

Variable	Categories	No disease(n = 1413)	TB a (PTB+ETB)(n = 102)	Other AIDS-definingdisease (n = 56)	Total (n = 1571)	p value^b^
Sex	Male	728 (51.5)	59 (57.8)	44 (78.6)	831 (52.9)	<0.001
	Female	685 (48.5)	44 (42.2)	12 (21.4)	740 (47.1)	–
Age (years)	<30	398 (28.2)	22 (21.6)	13 (23.2)	433 (27.6)	0.42
	30–39	608 (43.0)	53 (52.0)	29 (51.8)	690 (43.9)	–
	40–49	314 (22.2)	19 (18.6)	12 (21.4)	345 (22.0)	–
	> = 50	93 (6.6)	8 (7.8)	2 (3.6)	103 (6.5)	–
Country	Brazil	222 (15.7)	4 (3.9)	5 (8.9)	231 (14.7)	<0.001
	Haiti	100 (7.1)	0 (0.0)	0 (0.0)	100 (6.4)	–
	India	198 (14.0)	57 (55.9)	0 (0.0)	255 (16.2)	–
	Malawi	205 (14.5)	10 (9.8)	6 (10.7)	221 (14.0)	–
	Peru	116 (8.2)	3 (2.9)	15 (26.8)	134 (8.5)	–
	South Africa	181 (12.8)	27 (26.5)	2 (3.6)	210 (13.4)	–
	Thailand	92 (6.5)	0 (0.0)	8 (14.3)	100 (6.4)	–
	United States	191 (13.5)	0 (0.0)	19 (33.9)	210 (13.4)	–
	Zimbabwe	108 (7.7)	1 (1.0)	1 (1.8)	110 (7.0)	–
CD4 count (cells/mm^3^)	<50	170 (12.0)	12 (11.8)	18 (32.1)	200 (12.7)	<0.001
	50–99	190 (13.4)	27 (26.4)	12 (21.4)	229 (14.6)	–
	100–199	477 (33.8)	41 (40.2)	10 (17.9)	528 (33.6)	–
	200–299	576 (40.8)	22 (21.6)	16 (28.6)	614 (39.1)	–
Viral load (c/mL)	<100,000	704 (49.8)	26 (25.5)	25 (44.6)	755 (48.0)	<0.001
	≥100,000	709 (50.2)	76 (74.5)	31 (55.4)	816 (52.0)	–
Group allocation c	ZDV/3TC+ EFV	465 (32.9)	38 (37.2)	16 (28.6)	519 (33.0)	0.58
	DDI+FTC+ ATV	480 (34.0)	28 (27.5)	18 (32.1)	526 (33.5)	–
	FTC/TDF+ EFV	468 (33.1)	36 (35.3)	22 (39.3)	526 (33.5)	–

^a^ TB = tuberculosis; PTB = pulmonary tuberculosis; ETB = extrapulmonary tuberculosis.

^b^ p-values for categorical variables were obtained with the use of the chi-square test, while ANOVA test was used for continuous variables.

^c^ ZDV = zidovudine; 3TC = lamivudine; EFV = efavirenz; DDI = didanosine; TDF = tenofovir; FTC = emtricitabine.

**Figure 1:**
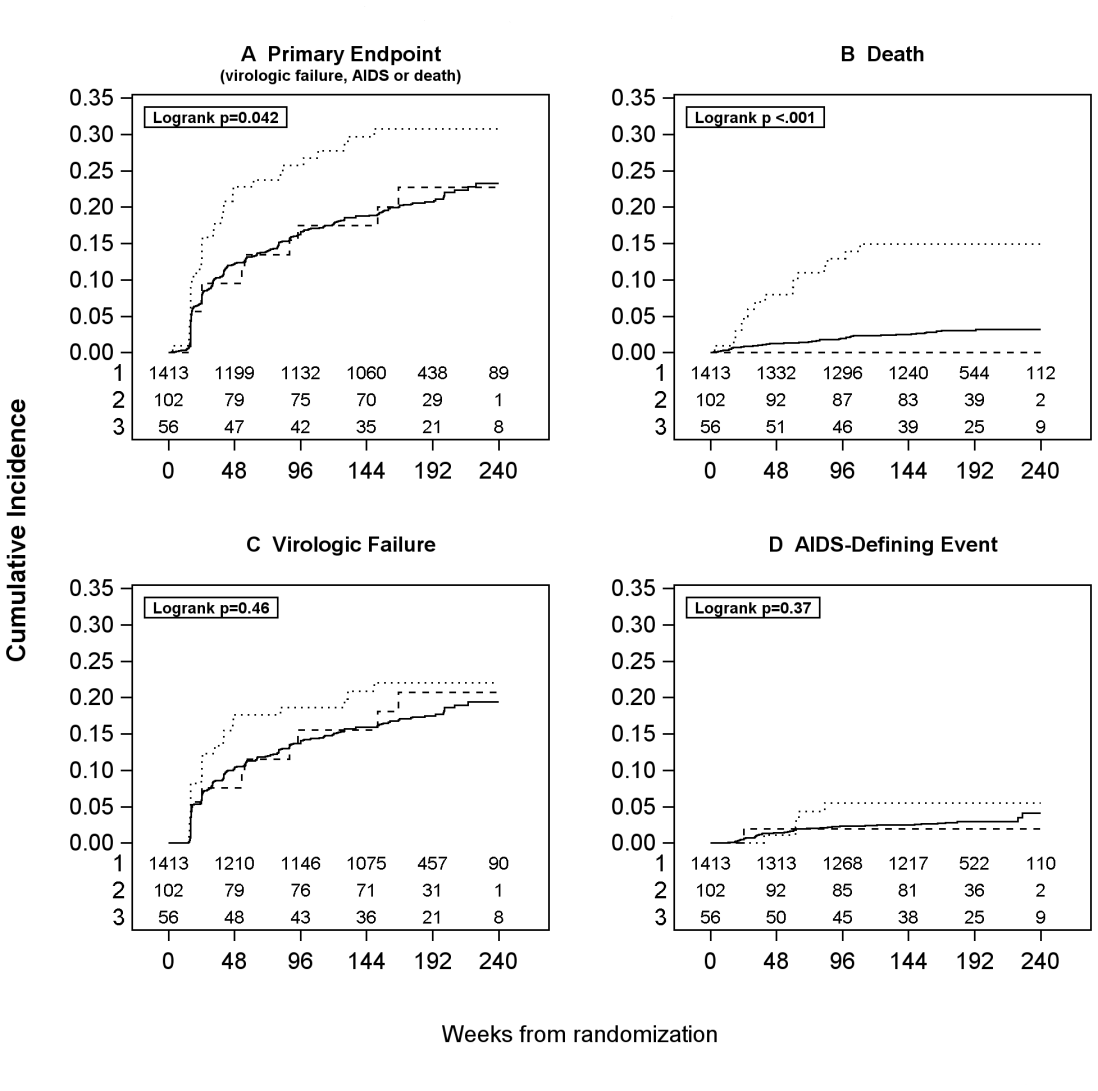
Outcomes according to study group. Groups: ____ No OI; …. TB; –– Other OIs.

Overall, 329 of the 1571 participants (21%) experienced a primary outcome event, including 287 (20%) in the “no disease” group, 31 (30%) in the “TB” group and 11 (20%) in the “other AIDS-defining disease” group (Table 2). Figure 1 shows the Kaplan-Meier curves for the proportion of participants without a primary outcome event over time for each of the three disease groups. There was a significant difference among the three groups (p = 0.042), reflecting a poorer outcome among participants in the “TB” group compared to the other two groups (Figure 1A).

**Table 2:** Summary of events and follow-up (censoring) times by study group.

Endpoints	Categories	No disease(n = 1413)	TB a(PTB+ETB) (n = 102)	OtherAIDS-definingdisease (n = 56)	Total(n = 1571)
Primary composite outcome b	Event – n(%)	287 (20.3)	31 (30.4)	11 (19.6)	329 (20.9)
	Median time of follow-up for outcomes (weeks)	176	178	168	–
Death	Event – n(%)	41 (2.9)	15 (14.7)	0 (0.0)	56 (3.6)
	Median time of follow-up for outcomes (weeks)	184	186	183	–
Virologic failure	Event – n(%)	239 (16.9)	21 (20.6)	10 (17.8)	270 (17.2)
	Median time of follow-up for outcomes (weeks)	177	179	169	–
New AIDS-definingdisease	Event – n(%)	40 (2.8)	5 (4.9)	1 (1.8)	46 (2.9)
	Median time of follow-up for outcomes (weeks)	184	184	176	–

^a^ TB = tuberculosis; PTB = pulmonary tuberculosis; ETB = extrapulmonary tuberculosis.

^b^ First of virologic failure, new AIDS-defining disease and death.

In the overall study population, 56 participants (4%) died, including 41 (3%) in the “no disease” group and 15 (15%) in the “TB” group (Table 2). Six deaths in the “TB group” were considered HIV-related (two PCP and one of each miliary TB, non-Hodgkin lymphoma, brain tuberculoma and chronic gastroenteritis), 7 were considered not related to HIV (three hepatitis and one of each stroke, acute myocardial infarction, hydrocephaly and stabbing) and two were not categorized (one cerebral hemorrhage and one suicide). No deaths were reported among the 56 participants in the “other AIDS-defining disease” group. There was a highly significant difference among the three study groups for the mortality outcome (p<0.001, Figure 1B), again reflecting a poorer outcome among participants in the “TB” group. Forty percent of deaths in the “TB” group were recorded as HIV related (PCP, ETB and lymphoma).

A total of 270 participants (17%) experienced virologic failure during follow-up, including 239 (17%) in the “no disease” group, 21 (21%) in the “TB” group and 10 (18%) in the “other AIDS-defining disease” group (Table 2). There was not a significant difference among the three groups in distribution of time to virologic failure (p = 0.46, Figure 1C). The proportion of participants experiencing AIDS-defining disease progression was comparatively low: 46 participants (3%) in the overall study population, including 40 (3%) in the “no disease” group, 5 (5%) in the “TB” group and 1 (2%) in the “other AIDS-defining disease” group, with no significant difference among the groups (p = 0.37, Figure 1D).

Table 3 provides results from univariate and multivariate analyses of the association between study group and risk of experiencing a composite primary outcome and each component of the primary outcome separately. In univariate analyses, the “TB” group was significantly associated with a higher risk of a composite primary outcome. In the multivariate model, using the group with no disease as reference, and adjusting for sex, age, country, CD4+ cell count, viral load and randomized ART assignment, an ongoing diagnosis of TB at randomization was associated with an increased risk of the primary outcome (adjusted hazard ratio [AHR] 1.38; 95%CI: 0.92–2.10) but this was not statistically significant (p = 0.11). There was no evidence that the differences among disease groups varied by ART assignment. When evaluating death as the primary outcome, the “TB” group was significantly associated with a higher risk of death in the univariate analysis. In the multivariate model which included sex, age, country, CD4+ cell count, viral load, ART assignment and the interaction term for sex and ART assignment, using the group with no disease as reference, an ongoing diagnosis of TB at randomization was associated with an increased risk of death (AHR 3.41; 95%CI: 1.72–6.75) and this association was highly significant (p<0.001). No significant association was found in analyses of time to virologic failure or time to AIDS-defining events.

**Table 3:** Univariate and multivariate analysis for the composite (first of virologic failure, AIDS defining event and all-cause mortality) primary outcome and for the separate components of the primary outcome by study group.

Parameter	Categories	UnadjustedHazard Ratio	95% Confidence Limits		p value	AdjustedHazard Ratio a	95% Confidence Limits		p value
Composite	No disease	Reference	–	–	–	Reference	–	–	–
	TB	1.60	1.11	2.32	0.013	1.39	0.93	2.10	0.11
	Other AIDS-defining disease	1.01	0.55	1.85	0.96	0.95	0.51	1.78	0.88
Death	No disease	Reference	–	–	–	Reference	–	–	–
	TB	5.29	2.93	9.53	<0.001	3.41	1.72	6.75	<0.001
	Other AIDS-defining disease	No estimate	.	.	.	No estimate	.	.	.
Virological failure	No disease	Reference	–	–	–	Reference	–	–	–
	TB	1.32	0.84	2.06	0.22	1.30	0.81	2.11	0.29
	Other AIDS-defining disease	1.10	0.58	2.07	0.77	1.06	0.55	2.04	0.86
AIDS-defining event	No disease	Reference	–	–	–	Reference	–	–	–
	TB	1,87	0.73	4.73	0.19	0.95	0.34	2.63	0.92
	Other AIDS-defining disease	0.66	0.09	4.84	0.68	0.49	0.06	3.77	0.49

^a^ Adjusted for age, sex, country, screening CD4, baseline viral load, chance imbalance by ART regimen and the interaction term sex*ART regimen.

^b^ No estimate available as there were no deaths during follow-up among participants in this category.

## Discussion

In univariate analyses, participants with ongoing TB at ART initiation were more likely to experience a primary outcome (virologic failure, AIDS-defining disease progression or death) compared with participants with no AIDS-defining disease (p = 0.013). This finding appeared to be driven by a higher risk of death (p<0.001). However, in multivariate analyses, although the increased risk of a primary outcome in the TB group was not statistically significant after controlling for confounding covariates such as sex, ART regimen assignment, age, screening CD4+ cell count and baseline viral load (p = 0.11), the risk of death remained significantly elevated (p<0.001). No death among participants in the TB group was associated with immune reconstitution inflammatory syndrome (IRIS). Of note, participants with TB did not have an increased risk of virological failure so it is not likely that they died as a result of ineffective ART. Among participants with other active AIDS-defining diseases (most commonly mucocutaneous herpes simplex or wasting syndrome), there was not a significant difference in risk of a primary outcome, or specifically of death, compared with participants with no active AIDS-defining disease.

In 2010, Straetemans et al. published a meta-analysis of cohort studies in which the authors evaluated outcomes among HIV-infected patients with and without TB, and concluded that co-infected patients had a higher risk of death compared to patients without TB [11]. The same was not true for studies that included patients receiving ART, in which no significant increase in risk was found [11], [21]. In 2011, Dronda et al. published the results of a cohort study in Spain in which the authors evaluated various outcomes in treatment naïve patients comparing those with a prior diagnosis of TB versus other AIDS-defining illness (ADI) to those without TB or ADI at ART initiation [22]. They concluded that a previous diagnosis of TB or ADI did not compromise the virological and immunological responses to treatment. However, contrary to our findings, the authors found that a prior diagnosis of an ADI other than TB, but not TB, was associated with an increased risk of death when compared with patients who started ART in the absence of a previous ADI. A difference in timing of ART initiation may explain the difference in results (the median time from TB diagnosis to ART initiation was 53 days in the Spanish cohort and approximately 100 days in our study) as timing of ART initiation in patients with TB has been shown to be an important predictor of good response [12]–[14]. Havlir *et al,* showed an improvement in survival in patients starting TB treatment immediately (within 2 weeks) versus early (8–12 weeks), especially among those with CD4+ cell counts below 50 cells/mm^3^ [15]. However, TB immune reconstitution inflammatory syndrome (IRIS) was more common with immediate ART. Despite the increased risk of death in patients starting ARV with TB co-infection, the risk could be higher without ARV treatment [15]. Such studies prompted the World Health Organization to update its guidelines and recommend early ART initiation, ideally between 2 and 8 weeks of starting TB treatment [16]. Our study lacked power to compare outcomes according to timing of ART initiation relative to TB diagnosis, and the optimum time for ART initiation is still open to debate [23], [24].

Herein, we have presented a *post hoc* analysis of data from a randomized clinical trial which was designed to evaluate the efficacy of three different ART regimens. However, this comparison of the three study groups according to the baseline occurrence of an AIDS-defining disease is observational and hence is subject to covariates that could confound the real association. This analysis did account for a number of the important confounding variables described in the literature and others that could be related to the study design such as country and randomized treatment assignment. The reason for increased risk of death in the “TB” group is unknown since our data show little mortality attributable to TB or specific AIDS related complications. The finding that non-tuberculosis AIDS diseases were not associated with greater mortality is somewhat surprising and may reflect the occurrence of less severe non-TB diseases among study participants. Although no restriction was made in the design of the clinical trial on the inclusion of patients with AIDS-defining diseases except that they should be on treatment and stable, it is possible that sites may have chosen patients with HIV infection in earlier stages to facilitate follow-up. The low number of AIDS-defining diseases in our groups may have resulted in lower power to detect differences among the groups and may explain the fact that our results for the TB group did not reach statistical significance in the multivariate analysis for the composite primary outcome. However, we still observed that the TB group difference is mostly driven by all-cause mortality, and these results were also significant in the multivariate analysis.

### Conclusion

Our results show that a diagnosis of active TB, but not AIDS-defining disease, at ART initiation was associated with an increased risk of morbidity and mortality in HIV-1 infected patients. Of note, the increased risk for death persisted when adjusted for important risk factors such as CD4+ cell count, viral load and ART regimen. Our results reinforce the potential importance of early HIV treatment in HIV-TB co-infection in order to increase survival. Campaigns to increase HIV awareness are essential to achieve earlier HIV testing prior to development of TB that may, in turn, improve long-term survival among TB patients. The data argue for earlier HIV diagnosis, but also the need for more research to further understand the unique risks and potential management strategies for those with TB at the time they initiate ART.
